# Complement C5a Receptor 1 Exacerbates the Pathophysiology of *N. meningitidis* Sepsis and Is a Potential Target for Disease Treatment

**DOI:** 10.1128/mBio.01755-17

**Published:** 2018-01-23

**Authors:** Johannes B. Herrmann, Marcel Muenstermann, Lea Strobel, Alexandra Schubert-Unkmeir, Trent M. Woodruff, Scott D. Gray-Owen, Andreas Klos, Kay O. Johswich

**Affiliations:** a Institute for Hygiene and Microbiology, University of Würzburg, Würzburg, Germany; b School of Biomedical Sciences, the University of Queensland, St. Lucia, Australia; c Department of Molecular Genetics, University of Toronto, Toronto, Canada; d Institute for Medical Microbiology and Hospital Epidemiology, Hannover Medical School, Hannover, Germany; GSK Vaccines

**Keywords:** C5aR1, *Neisseria meningitidis*, anaphylatoxins, complement system, inflammation, invasive disease, mouse model, neutrophils, sepsis, whole-blood model

## Abstract

Sepsis caused by *Neisseria meningitidis* (meningococcus) is a rapidly progressing, life-threatening disease. Because its initial symptoms are rather unspecific, medical attention is often sought too late, i.e., when the systemic inflammatory response is already unleashed. This in turn limits the success of antibiotic treatment. The complement system is generally accepted as the most important innate immune determinant against invasive meningococcal disease since it protects the host through the bactericidal membrane attack complex. However, complement activation concomitantly liberates the C5a peptide, and it remains unclear whether this potent anaphylatoxin contributes to protection and/or drives the rapidly progressing immunopathogenesis associated with meningococcal disease. Here, we dissected the specific contribution of C5a receptor 1 (C5aR1), the canonical receptor for C5a, using a mouse model of meningococcal sepsis. Mice lacking C3 or C5 displayed susceptibility that was enhanced by >1,000-fold or 100-fold, respectively, consistent with the contribution of these components to protection. In clear contrast, *C5ar1*^−/−^ mice resisted invasive meningococcal infection and cleared *N. meningitidis* more rapidly than wild-type (WT) animals. This favorable outcome stemmed from an ameliorated inflammatory cytokine response to *N. meningitidis* in *C5ar1*^−/−^ mice in both *in vivo* and *ex vivo* whole-blood infections. In addition, inhibition of C5aR1 signaling without interference with the complement bactericidal activity reduced the inflammatory response also in human whole blood. Enticingly, pharmacologic C5aR1 blockade enhanced mouse survival and lowered meningococcal burden even when the treatment was administered after sepsis induction. Together, our findings demonstrate that C5aR1 drives the pathophysiology associated with meningococcal sepsis and provides a promising target for adjunctive therapy.

## IMPORTANCE

The devastating consequences of *N. meningitidis* sepsis arise due to the rapidly arising and self-propagating inflammatory response that mobilizes antibacterial defenses but also drives the immunopathology associated with meningococcemia. The complement cascade provides innate broad-spectrum protection against infection by directly damaging the envelope of pathogenic microbes through the membrane attack complex and triggers an inflammatory response via the C5a peptide and its receptor C5aR1 aimed at mobilizing cellular effectors of immunity. Here, we consider the potential of separating the bactericidal activities of the complement cascade from its immune activating function to improve outcome of *N. meningitidis* sepsis. Our findings demonstrate that the specific genetic or pharmacological disruption of C5aR1 rapidly ameliorates disease by suppressing the pathogenic inflammatory response and, surprisingly, allows faster clearance of the bacterial infection. This outcome provides a clear demonstration of the therapeutic benefit of the use of C5aR1-specific inhibitors to improve the outcome of invasive meningococcal disease.

## INTRODUCTION

*Neisseria meningitidis* frequently inhabits the human upper respiratory tract (1). Colonization is usually asymptomatic, but *N. meningitidis* can cross the epithelial barrier and enter the bloodstream, where it survives and multiplies, thereby causing invasive meningococcal diseases (IMD) such as sepsis or meningitis (2). Meningococcal sepsis has a rapid onset with rather unspecific initial symptoms and primarily affects infants and toddlers (3). This complicates timely diagnosis before severe symptoms occur, including disseminated intravascular coagulopathy, hypovolemia, shock, loss of consciousness, and multiorgan failure (2–4). Current regimens of treatment of meningococcal sepsis include prompt administration of antibiotics and aggressive fluid management to maintain circulation and organ perfusion (5).

The ability of *N. meningitidis* to survive in blood is chiefly attributable to its polysaccharide capsule, which protects against host immune functions such as complement and phagocytosis (6). Conversely, the complement system is the primary innate immune determinant against IMD (7). Its activation in bacterial infections generally serves three main purposes: opsonization for phagocytosis through the activity of C3b, inflammation for phagocyte recruitment through the activity of C3a and C5a, and bacteriolysis via the membrane attack complex (MAC). A schematic of complement activation is depicted in Fig. S1 in the supplemental material.

10.1128/mBio.01755-17.1 FIG S1  Schematic of the complement cascade. Download FIG S1, PDF file, 0.4 MB. Copyright © 2018 Herrmann et al. 2018 Herrmann et al. This content is distributed under the terms of the Creative Commons Attribution 4.0 International license.

The lytic pathway is of particular importance to IMD, as individuals with deficiencies in the late complement components (C5 to C9) face a several-thousand-fold-increased risk of contracting IMD (6). The magnitude of this association and the fact that *N. meningitidis* vaccines are benchmarked by their efficacy in inducing bactericidal antibodies capable of targeting complement to *N. meningitidis* for lysis (8) have drawn most research attention toward the lytic pathway in the interplay between *N. meningitidis* and the complement system. Curiously, the course of disease in these patients appears to be less fulminant than in complement-sufficient ones, suggesting an inverse link between protection and pathogenesis (6).

While they are not directly involved in bacterial opsonization or MAC-dependent killing, C3a and C5a are released during the complement cascade. These anaphylatoxin peptides have drawn little attention in the context of meningococcal infection but have been implicated in various inflammatory disorders, including asthma, ischemia-reperfusion injury, autoimmune diseases, inflammatory bowel diseases, and sepsis (9). C5a induces a more profound proinflammatory response than C3a (10) and is a strong chemoattractant for cells of the myeloid lineage as a consequence of triggering C5a receptor 1 (C5aR1) (11). C5aR1 is a G-protein-coupled receptor highly expressed by granulocytes (11, 12), but it is also found on monocytes, macrophages, dendritic cells, and mast cells and on nonimmune cells such as endothelial cells, cardiomyocytes, astrocytes, and others (13–15). C5aR1 activation triggers chemotaxis, degranulation, and oxidative burst in granulocytes (11). Due to the pleiotropic effects exerted by the C5a/C5aR1 axis, its activation during infectious diseases entails different outcomes. For example, C5aR1 activation is pivotal for efficient pathogen removal in cases of *Pseudomonas aeruginosa* lung infection (16), while C5aR1 fuels systemic hyperinflammation in polymicrobial sepsis after cecum ligation and puncture (CLP) and is detrimental to the host (17). Here, we used a mouse model of *N. meningitidis* sepsis to understand the respective contributions of the different functional outputs of the complement cascade. Our findings highlight the detrimental role of C5aR1 and uncover its potential as a therapeutic target to ameliorate IMD outcomes in an *ex vivo* human blood model and *in vivo* in mice.

## RESULTS

### Early and late steps in the complement cascade are essential for protection in a murine model of *N. meningitidis* sepsis.

**FIG 1:**
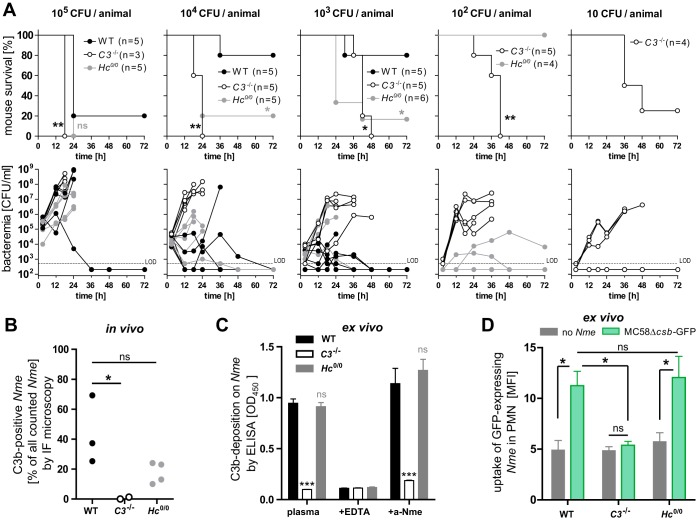
Role of complement in murine model of *N. meningitidis* sepsis. (A) Survival curves (top panels) and bacteremia (lower panels) of WT, C3^−/−^, and *Hc*°^/^° mice intraperitoneally infected with *N. meningitidis* strain MC58 inocula as indicated above the graphs. LOD, limit of detection; ns, not significant; *, *P* < 0.05; **, *P* < 0.01 (in Mantel-Cox test). (B) *In vivo* C3b deposition onto *N. meningitidis* (*Nme*) in infected mice. Blood smears of the infected mice described in the panel A legend were analyzed by immunofluorescence microscopy at ×60 magnification after staining with DAPI, rabbit anti-*N. meningitidis* (Alexa 488 channel), and anti-C3 (Cy3 channel). About 500 *N. meningitidis* cells per sample were identified by green fluorescence, and the fraction of C3-positive *N. meningitidis* cells was expressed as the percentage of all analyzed *N. meningitidis* cells. Each dot represents results from one animal. Representative immunofluorescence (IF) microscopy images are shown in Fig. S3. ns, not significant; *, *P* < 0.05 (in one-way analysis of variance [ANOVA] with Dunnett’s *post hoc* test). (C) *Ex vivo* C3b deposition from mouse lepirudin plasma on *N. meningitidis* MC58 as analyzed by whole-cell ELISA. ns, not significant; ***, *P* < 0.005 (in one-way ANOVA with Dunnett’s *post hoc* test). (D) *Ex vivo* uptake of *N. meningitidis* by neutrophils of WT, C3^−/−^, and *Hc*°^/^° mice. Lepirudinized whole-mouse blood was infected with 10^7^ CFU/ml of acapsulate *N. meningitidis* expressing GFP (MC58Δ*csb*-GFP), and the mean fluorescence intensity (MFI) of neutrophils (PMN; gated as Ly6G^hi^) was analyzed by flow cytometry. The graph shows means ± standard deviations of the means (SEM) of results from three independent experiments. *, *P* < 0.05 (in one-way ANOVA with Bonferroni’s *post hoc* test). ns, not significant.

A functional complement system is vital for the defense against IMD (6); however, no study has yet directly compared the *in vivo* contributions of the different branches of the complement effector mechanisms to *N. meningitidis* immunity and immunopathology. Therefore, we analyzed the contribution of C3- and C5-dependent complement activities in a mouse model of *N. meningitidis* sepsis after intraperitoneal (i.p.) infection by comparing wild-type (WT) mice with C3^−/−^ mice (completely devoid of complement effector functions) and *Hc*°^/^° mice lacking C5 (allowing C3b opsonization but devoid of MAC and C5a formation). Although humans represent the only natural host of these bacteria (18), several mouse models have been developed that allow *in vivo* investigation of IMD (19–21) as well as colonization (22, 23). Congenic *Hc*°^/^° mice were obtained by 10 generations of backcrossing of the Hc° allele from FVB mice onto the C57BL/6J background. Phenotypic characterization of the B6.FVB-*Hc*° mice obtained (termed *Hc*°^/^° here) in relation to the other, established mouse strains on the C57BL/6J genetic background used in this study is shown in Fig. S2 in the supplemental material. To quantify the differential effects of C3 deficiency versus C5 deficiency, mice received different inocula of prototypic *N. meningitidis* serogroup B strain MC58 and their survival and bacterial burden were assessed over time (Fig. 1A). C3-deficient mice succumbed to an inoculum of as little as 10 CFU of *N. meningitidis*, while 1,000 CFU was the lowest lethal dose for *Hc*°^/^° mice and 10^5^ CFU was lethal for WT mice. The different levels of susceptibility of these mouse lines correlated with their levels of meningococcemia, implying that the C3- and C5-deficient animals were defective in their ability to clear the infection.

10.1128/mBio.01755-17.2 FIG S2  Characterization of *Hc*°^/^° mice. Download FIG S2, PDF file, 0.2 MB. Copyright © 2018 Herrmann et al. 2018 Herrmann et al. This content is distributed under the terms of the Creative Commons Attribution 4.0 International license.

In order to further investigate the contribution of opsonophagocytosis to *N. meningitidis* clearance, we analyzed whether *N. meningitidis* was indeed opsonized during *in vivo* infection. Immunofluorescence staining of blood smears indicated that meningococci are indeed efficiently opsonized with C3b during infection in WT mice and in *Hc*°^/^° mice, whereas no opsonization occurred in C3^−/−^ mice (Fig. 1B; see also Fig. S3). This C3b deposition pattern was also evident in an *ex vivo* assay performed with mouse plasma (Fig. 1C) subjected to anticoagulation with lepirudin, an anticoagulant that does not interfere with the complement system (24). Consistent with these observations, neutrophil uptake of green fluorescent protein (GFP)-expressing meningococci (MC58Δ*csb*-GFP) occurred at comparable levels in WT and *Hc*°^/^° mice, whereas neutrophils from C3^−/−^ mice showed no *N. meningitidis* uptake, as assayed by flow cytometry in a lepirudinized whole-blood model *ex vivo* (Fig. 1D). Thus, opsonophagocytosis has a significant impact on experimental disease outcome, as evidenced by the ≥100-fold-higher susceptibility of C3^−/−^ mice than *Hc*°^/^° mice. While the *Hc*°^/^° mice do have normal opsonophagocytic activity, they are still ~100-fold more susceptible than WT mice, highlighting the multidimensional protection afforded by the complement cascade. However, since C5 deficiency blunts both the bactericidal (MAC) and inflammatory (C5a) effects of complement, the specific contribution of each cannot be determined with these animals.

10.1128/mBio.01755-17.3 FIG S3  Representative immunofluorescence images for graph shown in Fig. 1B. Download FIG S3, PDF file, 2.1 MB. Copyright © 2018 Herrmann et al. 2018 Herrmann et al. This content is distributed under the terms of the Creative Commons Attribution 4.0 International license.

### Anaphylatoxins C3a and C5a are released during experimental *N. meningitidis* sepsis.

**FIG 2:**
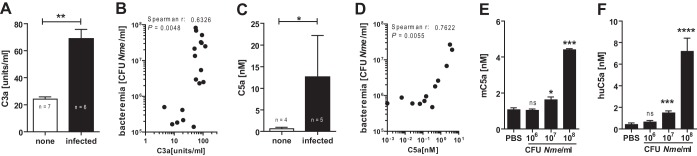
Anaphylatoxin release during *N. meningitidis* sepsis. (A and C) Plasma levels of C3a and C5a, respectively, in WT mice at 12 h postinfection with 10^5^ CFU of *N. meningitidis* strain MC58 as measured by ELISA. Plotted are means ± SEM. *, *P* < 0.05; **, *P* < 0.01 (in unpaired, two-tailed Student’s *t* test). (B and D) Correlation of C3a and C5a concentrations, respectively, with bacterial burden of infected mice. (E and F) Dose response of plasma C5a liberation in *ex vivo* infection of lepirudin anticoagulated whole-mouse blood (*n* = 3 independent samples) and whole human blood (*n* = 15 individual donors), respectively, with *N. meningitidis* MC58 (means ± SEM). *, *P* < 0.05; ***, <0.001; ****, <0.0001 (in one-way ANOVA with Dunnett’s *post hoc* test using PBS as a comparator).

Next, we attempted to assess the influence of complement-derived inflammation. The opsonization of *N. meningitidis* (Fig. 1) indicates that complement is activated during the course of infection, which should liberate the C3a and C5a anaphylatoxins. Indeed, plasma levels of both mediators were significantly enhanced upon infection, with C3a levels increasing 3-fold and C5a levels 12-fold compared to the levels seen with uninfected mice (Fig. 2A and C) and in both cases correlating strongly with bacterial burden during the course of disease (Fig. 2B and D). To dissect the contribution of C5 bactericidal and inflammatory effects, and since C5a is substantially more potent in triggering inflammation than C3a, further analysis focused solely on C5a. *Ex vivo* experiments using lepirudin-anticoagulated mouse blood (Fig. 2E) indicated dose dependency of the C5a release, and this reflected the response in human blood (Fig. 2F). Since many pathogenic traits vary among *N. meningitidis* strains, we also considered potential differences among different isolates and found robust C5a liberation in whole blood for all strains of an isolate collection covering the most relevant *N. meningitidis* serogroups and sequence types in mouse whole blood (Fig. S4A) and also in human whole blood (Fig. S4B).

10.1128/mBio.01755-17.4 FIG S4  C5a liberation in whole-blood models of mouse and human by different *N. meningitidis* strains. Download FIG S4, PDF file, 0.2 MB. Copyright © 2018 Herrmann et al. 2018 Herrmann et al. This content is distributed under the terms of the Creative Commons Attribution 4.0 International license.

### *C5ar1*-deficient mice display significantly ameliorated *N. meningitidis* sepsis.

**FIG 3:**
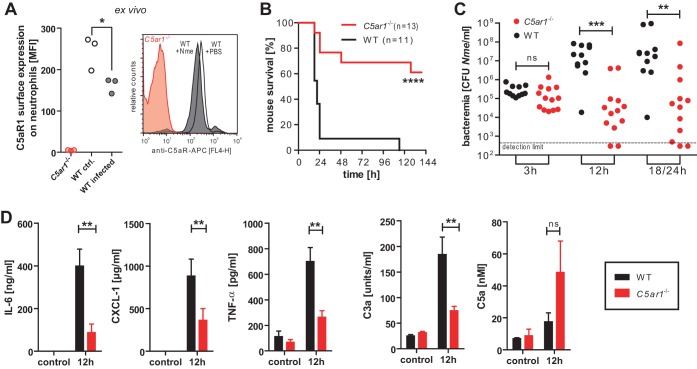
*In vivo N. meningitidis* sepsis in WT versus C5ar1^−/−^ mice. (A) Surface expression of C5aR1 as measured by flow cytometry on neutrophils (gated as Ly6G^hi^) in WT mouse lepirudin-treated whole blood after 1 h of infection with 10^7^ CFU of *N. meningitidis* MC58 versus uninfected control (ctrl.) and *C5ar1*^−/−^ neutrophils as a staining control. Plotted is the mean fluorescence intensity (MFI) of results from three independent experiments. *, *P* < 0.05 (in paired matched observations per mouse; two-tailed Student’s *t* test). (B) Survival curves of WT and C5aR1^−/−^ mice after intraperitoneal infection with 10^5^ CFU of *N. meningitidis* MC58. ****, *P* < 0.0001 (in Mantel-Cox test). (C) *N. meningitidis* counts in blood of infected mice at indicated time points. The 18/24 h data comprise 18-h values from mice not surviving until 24 h plus 24-h values from the mice surviving until then. ns, not significant; **, *P* < 0.001; ***, *P* < 0.0001 (in unpaired, two-tailed Mann-Whitney test). (D) Plasma levels of inflammatory mediators at 12 h after intraperitoneal infection with 10^5^ CFU *N. meningitidis* MC58 of WT versus *C5ar1*^−/−^ mice (means ± SEM; *n* = 5 per genotype). ns, not significant; **, *P* < 0.01 (in unpaired, two-tailed Student’s *t* test).

Since C5a levels were markedly increased during experimental *N. meningitidis* sepsis, we sought to assess the specific contribution of this potent anaphylatoxin by determining the specific contribution of C5aR1, the canonical cellular receptor by which C5a expresses its inflammatory potential. When C5a activates C5aR1, the C5a/C5aR1 complex is rapidly internalized, thereby reducing surface C5aR1 levels on neutrophils (25); this phenomenon has been linked to poor outcome in sepsis patients as well as in rodent models of sepsis (26, 27). To confirm that C5aR1 is engaged during meningococcal infection, lepirudinized whole blood from mice was infected *ex vivo* with *N. meningitidis* and surface expression of C5aR1 on neutrophils was assessed by flow cytometry (Fig. 3A). The resultant reduction of C5aR1 on WT neutrophils indicates that the C5a liberated during *N. meningitidis* infection in this model indeed triggered C5aR1 activation and subsequent cellular internalization. Next, we assessed the specific contribution of C5aR1 during *N. meningitidis* sepsis by comparing the outcomes seen with systematic infections of WT and *C5ar1*^−/−^ mice. In clear contrast to the response of C5-deficient mice (Fig. 1), the mice lacking C5aR1 displayed significantly higher survival rates (Fig. 3B) and lower levels of bacteremia (Fig. 3C) than WT mice under conditions of infection with 10^5^ CFU. Furthermore, plasma levels of inflammatory mediators interleukin-6 (IL-6), CXCL-1, and tumor necrosis factor (TNF) were significantly lower in C5ar1^−/−^ mice than in WT mice at 12 h of infection (Fig. 3D). The increased generation of C3-derived C3a in WT mice versus C5ar1^−/−^ mice presumably reflects the heightened complement activation due to the increased bacterial burden at 12 h. Conversely, C5a levels were higher in C5ar1^−/−^ mice despite their significantly lower meningococcal burden; this is consistent with the established role of C5aR1 in C5a sequestration (25). Notably, all mice succumbed to higher *N. meningitidis* doses (10^6^ or 10^7^ CFU) independently of the genotype in spite of a nonsignificant trend for lower bacteremia in *C5ar1*^−/−^ mice (Fig. S5). Taken together, these results indicate that C5aR1 potently augments the pathophysiology associated with experimental *N. meningitidis* sepsis.

10.1128/mBio.01755-17.5 FIG S5  Survival and bacteremia in WT versus *C5ar1*^−/−^ mice after infection with different inocula of *N. meningitidis*. Download FIG S5, PDF file, 0.1 MB. Copyright © 2018 Herrmann et al. 2018 Herrmann et al. This content is distributed under the terms of the Creative Commons Attribution 4.0 International license.

### Role of neutrophils and macrophages in experimental *N. meningitidis* sepsis.

**FIG 4:**
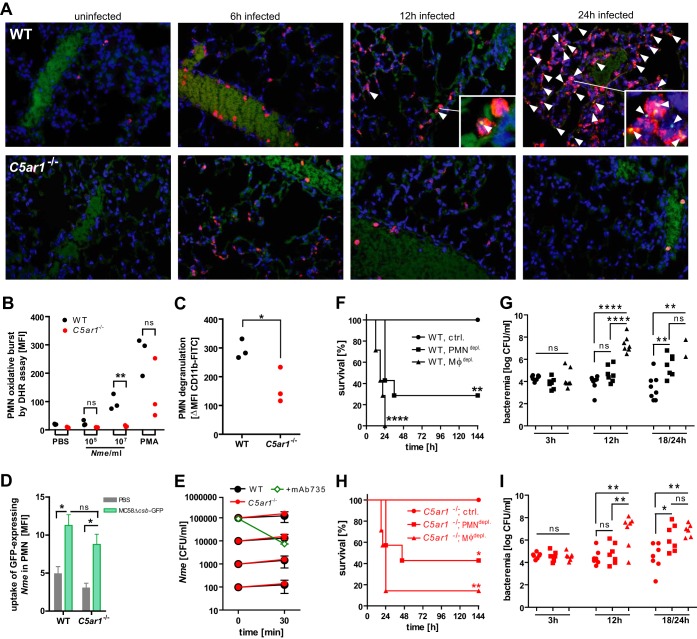
Role of phagocytes in *N. meningitidis* sepsis in WT and *C5ar1*^−/−^ mice. (A) Immunofluorescence microscopy (×60 magnification) of tissue sections of lung at indicated time points after intraperitoneal infection with 10^5^ CFU *N. meningitidis* MC58. Blue, nuclei; red, neutrophil elastase; green, *N. meningitidis* (arrowheads). Background data in the green channel stem from erythrocytes, indicating the position of blood vessels. Insets are enlargements of points of interest from the same image. (B) Oxidative burst of Ly6G^hi^ neutrophils (*n* = 3) assayed by DHR123 assay in lepirudin-treated whole blood infected with 10^5^ or 10^7^ CFU per ml of *N. meningitidis* MC58. The positive control was PMA (100 nM). ns, not significant; **, *P* < 0.01 (in unpaired, two-tailed Student’s *t* test). (C) Neutrophil degranulation measured as the difference in levels of CD11b surface expression between infected (10^7^ CFU/ml) and uninfected lepirudin-treated whole-blood samples from WT and *C5ar1*^−/−^ mice after 1 h of incubation. Neutrophils were gated as Ly6G^hi^ cells and CD11b stained with clone M1/70. *, *P* < 0.05 (in unpaired, two-tailed Student’s *t* test). (D) Uptake of *N. meningitidis* MC58Δ*csb*-GFP by Ly6G^hi^ neutrophils in *ex vivo* infection of lepirudin-treated whole blood (means ± SEM of the geometric mean of GFP fluorescence; *n* = 3). ns, not significant, *, *P* < 0.05 (in one-way ANOVA with Bonferroni’s *post hoc* test). (E) *Ex vivo N. meningitidis* survival at different inocula in lepirudin-treated whole blood of WT and C5ar1^−/−^ mice (means of CFU per milliliter ± SEM; *n* = 3). As a positive control for *N. meningitidis* killing, 1 µg/ml of anti-serogroup B mouse monoclonal antibody mAb735 was added. (F and H) Survival of *n* = 7 to 8 WT and *C5ar1*^−/−^ mice, respectively, infected with 10^4^ CFU of *N. meningitidis* MC58 after depletion of monocytes/macrophages (clodronate liposomes) or neutrophils (RB6-8C5) or the control (PBS). The experiment was conducted in a blind manner for depletion treatment. *, *P* < 0.05; **, *P* < 0.01; ***, *P* < 0.001; ****, *P* < 0.0001 (in Mantel-Cox analysis relative to control). (G and I) *N. meningitidis* counts in blood of mice in panels G and I. ns, not significant; *, *P* < 0.05; **, *P* < 0.01; ****, *P* < 0.0001 (in one-way ANOVA, applying Bonferrroni’s *post hoc* test).

Neutrophils are centrally important for bacterial clearance and are highly sensitive to C5a due to their robust expression of C5aR1 (28). However, during sepsis, neutrophils are strongly recruited to the lungs, contributing to acute respiratory distress syndrome (29). Indeed, in our infection model, we found intense neutrophil recruitment to the lungs of infected WT animals at 12 h and 24 h after infection with 10^5^ CFU *N. meningitidis* (Fig. 4A). Strikingly, this effect was only transient in C5ar1^−/−^ mice, implicating a C5a-dependent link in this pathogenic neutrophil response (Fig. 4A). Similarly, a strong mobilization of neutrophils was also evident in WT but not in C5ar1^−/−^ spleen and liver (Fig. S6A and B). This differential tissue distribution of neutrophils was particularly surprising because we observed that the density of neutrophils in blood rose comparably in the two mouse genotypes upon *N. meningitidis* infection (Fig. S6C), suggesting that the C5aR1-dependent recruitment of neutrophils into the organs was not due to differential induction of neutrophil development.

10.1128/mBio.01755-17.6 FIG S6  Neutrophil recruitment during *N. meningitidis* sepsis. Download FIG S6, PDF file, 0.1 MB. Copyright © 2018 Herrmann et al. 2018 Herrmann et al. This content is distributed under the terms of the Creative Commons Attribution 4.0 International license.

In order to determine how C5aR1 engagement influences the neutrophil response to *N. meningitidis*, we next conducted *ex vivo* infections with lepirudinized mouse whole blood. Compared to WT neutrophils, *C5ar1*^−/−^ neutrophils showed a pattern of significantly reduced neutrophil activation during *N. meningitidis* infection with respect to both the oxidative burst response, analyzed with the DHR123 assay (Fig. 4B), and degranulation, as measured by the increase in CD11b surface localization (Fig. 4C). However, the levels of *N. meningitidis* uptake were similar in *C5ar1*^−/−^ and WT neutrophils, as assayed by flow cytometry using an inoculum of GFP-expressing, unencapsulated *N. meningitidis* (Fig. 4D). Notably, the differences in neutrophil activation patterns did not impact the overall capability of *N. meningitidis* to survive and actually multiply in mouse whole blood *ex vivo*, even at low inoculation doses (Fig. 4E). Thus, the neutrophil defect in C5aR1 suppresses their oxidative burst and degranulation response to *N. meningitidis*, which may impact both the release of soluble bactericidal effectors and intercellular binding events, including extravasation, but the bacteria may still be effectively engulfed by these professional phagocytes.

The fact that *N. meningitidis* was able to proliferate in whole blood despite phagocytosis by polymorphonuclear leukocytes (PMNs) led us to analyze the specific contributions of the different phagocyte populations during meningococcal sepsis in mice. Therefore, neutrophils were depleted in WT and C5ar1^−/−^ mice by injection of antibody RB6-8C5 or monocytes and macrophages were depleted using clodronate liposomes prior to administering a sublethal dose of *N. meningitidis* (10^4^ CFU). All infected mice without depletion (receiving phosphate-buffered saline [PBS] instead) survived infection, whereas depletion of neutrophils and, particularly, monocytes/macrophages significantly increased mortality and bacteremia (Fig. 4F to I). Comparing the mouse genotypes, there was a nonsignificant trend to higher survival rates in neutrophil- or monocyte/macrophage-depleted *C5ar1*^−/−^ mice than in the corresponding treatment cohorts in WT mice; however, the overall results from the two mouse strains were very similar. This indicates that neutrophils and especially monocytes/macrophages are vital in the defense against *N. meningitidis* sepsis and that the antibacterial properties of these cells are not outweighed by concomitant deleterious effects of C5aR1 on these cells. Together, the data suggest that phagocytic populations significantly impact the course of experimental *N. meningitidis* sepsis.

### C5aR1 impacts inflammatory cytokine release *in vivo* and *ex vivo*.

**FIG 5:**
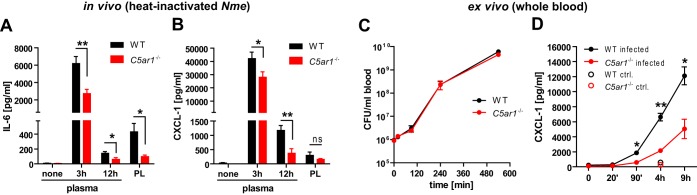
Cytokine induction in *in vivo N. meningitidis* infection of WT and *C5ar1*^−/−^ mice and in whole-blood model. (A and B) Levels of IL-6 and CXCL-1 in plasma and peritoneal lavage fluid (PL), respectively, from *n* = 8 mice per genotype after intraperitoneal administration of 5 × 10^8^ CFU of heat-inactivated *N. meningitidis* MC58. Plotted are means ± SEM. ns, not significant; *, *P* < 0.05; **, *P* < 0.01 (in unpaired, two-tailed Student’s *t* test). (C) Bacterial counts over time in *ex vivo* infection of lepirudin-treated mouse whole blood with 10^6^ CFU/ml *N. meningitidis*. Plotted are means ± SEM of results from *n* = 3 independent experiments. (D) CXCL-1 concentrations (means ± SEM) in plasma of whole-blood infection from the experiment described for panel C. *, *P* < 0.05; **, *P* < 0.01 (in unpaired, two-tailed Student’s *t* test).

WT mice showed an aggravated course of *N. meningitidis* sepsis, including a higher bacterial burden and also higher levels of inflammatory mediators in the blood than were seen with C5ar1^−/−^ mice (Fig. 3C and D). Higher bacterial loads stimulate stronger inflammatory responses, but uncontrolled inflammation can lead to immune paralysis (17), which in turn promotes bacterial escape from clearance and increases the bacterial burden; this interrelationship between inflammation and bacteremia makes it difficult to separate cause and consequence. In order to circumvent this problem, mice were injected with a heat-inactivated *N. meningitidis* inoculum and the cytokine response was assessed. Indeed, C5ar1^−/−^ mice displayed significantly lower plasma levels of the prototypic proinflammatory mediators IL-6 and CXCL-1 than WT mice in this setting (Fig. 5A and B). This finding was further corroborated in lepirudin-treated whole blood, in which viable *N. meningitidis* displayed identical growth kinetics (Fig. 5C) but induced significantly lower CXCL-1 levels in C5ar1^−/−^ mice (Fig. 5D). Therefore, C5aR1 drives the initial inflammatory cytokine response.

### C5aR1 blockade ameliorated cytokine release and neutrophil responses in a human whole-blood model of *N. meningitidis* sepsis.

**FIG 6:**
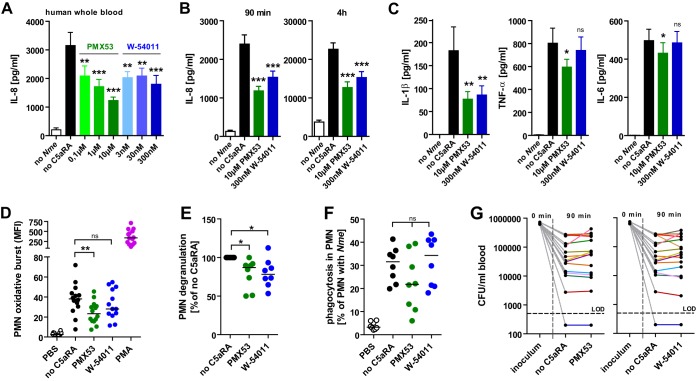
Inhibition of inflammatory cytokine release and neutrophil responses by C5aR1 blockade in lepirudin-anticoagulated human whole-blood infection with *N. meningitidis*. (A) IL-8 in human whole blood infected with 10^6^ CFU/ml *N. meningitidis* MC58 in the presence of C5aR1 antagonist (C5aRAs) PMX53 or W-54011 (means ± SEM; *n* = 5 donors). (B) IL-8 in human whole blood infected for the indicated durations with 10^6^ CFU/ml *N. meningitidis* MC58 in the presence of PMX53 (10 µM) or W-54011 (300 nM) (means ± SEM; *n* = 15 donors). (C) Cytokines at 90 min of infection of human whole blood with 10^6^ CFU *N. meningitidis* MC58 (means ± SEM; *n* = 16 donors). (D) Oxidative burst measured by DHR123 fluorescence in neutrophils during infection of whole human blood. (E) Neutrophil degranulation during whole-blood infection by surface localization of CD11b normalized to “no C5aRA.” (F) Phagocytosis of MC58-GFP by PMNs in whole blood as determined by flow cytometry and expressed as percentages of PMNs with an increase in the level of FL1-H above the level measured for the noninfected control. (G) *N. meningitidis* viability in blood of donors with or without C5aRAs. (D to F) Infection with 10^7^ CFU/ml of *N. meningitidis* MC58; lines indicate medians. (D to G) PMX53 was used at 10 µM and W-54011 at 300 nM. (A–G) *, *P* < 0.05; **, *P* < 0.01; ***, *P* < 0.001 (in repeated-measure ANOVA; matched observations per individual donor).

In order to test the feasibility of exploiting the protective effect of C5aR1 blockade for therapy in humans, we conducted infection experiments in lepirudinized whole blood from healthy volunteers. In agreement with the lower levels of proinflammatory cytokines and chemokines found in *C5aR1*^−/−^ mice (Fig. 5), the inflammatory response to *N. meningitidis* infection, as measured by IL-8 levels, declined in a dose-dependent manner in the presence of the peptide C5aR1 antagonist (C5aRA) PMX53 as well as with the nonpeptidic C5aRA W-54011 (Fig. 6A). This reduction was evident at both 90 min and 4 h of *ex vivo* infection (Fig. 6B) and was observed with all tested *N. meningitidis* strains (Fig. S7A). In addition to the IL-8 results, blockade of C5aR1 by PMX53 also significantly reduced *N. meningitidis*-induced IL-1β, TNF-α, and IL-6 levels, whereas W-54011 treatment reduced only the IL-1β levels (Fig. 6C). The levels of neutrophil oxidative burst (Fig. 6D) and degranulation (Fig. 6E) in response to *N. meningitidis* MC58 were significantly lowered by PMX53 treatment, whereas phagocytosis of MC58-GFP was not significantly affected (Fig. 6F). Testing the oxidative burst response to a variety of *N. meningitidis* strains, a trend similar to that seen with MC58 was observed, and the results reached significance only for strain Z2491 (Fig. S7B); this weaker effect seen with C5aRAs was most likely due to a smaller cohort having been tested (*n* = 4). Notably, the levels of *N. meningitidis* viability were similar in whole blood with or without C5aR antagonists (Fig. 6G), implying the absence of a defect in direct bacterial clearance attributable to these inhibitors. Thus, our data suggest that C5ar1 blockade ameliorates the inflammatory cytokine response in human whole blood.

10.1128/mBio.01755-17.7 FIG S7  IL-8 secretion and oxidative burst in human whole blood infected with various *N. meningitidis* strains. Download FIG S7, PDF file, 0.4 MB. Copyright © 2018 Herrmann et al. 2018 Herrmann et al. This content is distributed under the terms of the Creative Commons Attribution 4.0 International license.

### C5aR1 blockade ameliorated the course of experimental *N. meningitidis* sepsis.

**FIG 7:**
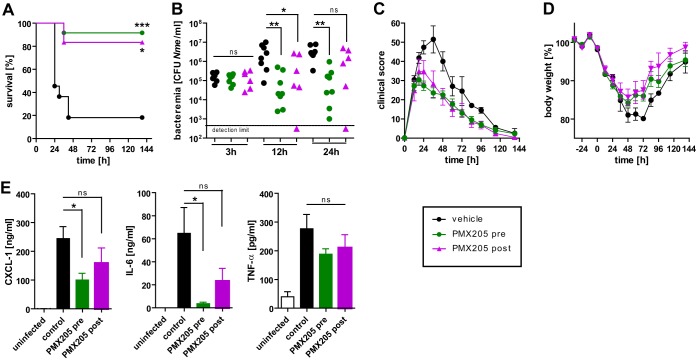
Pharmacologic targeting of C5aR1 ameliorates *in vivo N. meningitidis* sepsis. (A) Survival of WT mice after intraperitoneal infection with 10^5^ CFU of *N. meningitidis* strain MC58. Mice were randomized into three treatment cohorts with intraperitoneal injections every 6 to 12 h (see Materials and Methods) with 3 mg/kg with C5aR1-antagonist PMX205 starting either 12 h before infection (“pre”; *n* = 8) or 4 h after infection (“post”; *n* = 6) or received vehicle only (“control”; *n* = 8). The experiment was conducted in a blind manner with respect to treatment cohorts. *, *P* < 0.05; ***, *P* < 0.001 (in Mantel-Cox test). (B) Bacteremia in the mice from the experiment described for panel A. ns, not significant; *, *P* < 0.05; **, *P* < 0.01 (in Kruskal-Wallis test with Dunn’s *post hoc* test). (C) Clinical scores for mice from the experiment described for panel A over time. Plotted are means ± SEM. (D) Relative body weights (means ± SEM). (E) Levels of CXCL-1, IL-6, and TNF-α in tail vein blood samples from mice from the experiment described for panel A at 12 h. ns, not significant; *, *P* < 0.05 (in one-way ANOVA, applying Dunnett’s *post hoc* test with the control as the comparator).

Since *C5ar1* deficiency ameliorated the course of experimental *N. meningitidis* sepsis (Fig. 3) and its blockade also lowered the proinflammatory cytokine/chemokine levels in a human whole-blood infection model (Fig. 6), we speculated that C5aR1 might therefore represent a potential target for treatment. Since peptidic C5aR1 antagonist PMX53 showed a stronger therapeutic potential in human whole blood, we used a derivative specific for mouse C5aR1, PMX205 (cyclic hexapeptide hydro-cinnamate-[l-ornithine-proline-d-cyclohexylalanine-tryptophan-arginine]), which has previously been shown to block C5a-mediated inflammatory responses in multiple mouse models (30–33). Treatment of WT mice with PMX205 (3 mg/kg of body weight, intraperitoneal) was commenced either 12 h before infection (“pretreatment”) or 4 h after infection (“posttreatment”), whereas control animals received vehicle only. Both treatment regimens resulted in significantly higher survival rates, lower levels of bacteremia, less-severe clinical scores, a faster regain of body weight, and lower inflammatory cytokine levels than were seen with mice receiving vehicle control (Fig. 7). A bactericidal effect of PMX205 on *N. meningitidis* was ruled out by incubation of the bacteria in mouse serum spiked with increasing concentrations of the drug; the results showed no effect (Fig. S8). Therefore, therapeutic blockade of C5aR1 has a tremendous benefit by significantly improving the outcome of *N. meningitidis* sepsis even when it is administered after the onset of disease.

10.1128/mBio.01755-17.8 FIG S8  No bactericidal activity of PMX205 toward *N. meningitidis*. Download FIG S8, PDF file, 0.2 MB. Copyright © 2018 Herrmann et al. 2018 Herrmann et al. This content is distributed under the terms of the Creative Commons Attribution 4.0 International license.

## DISCUSSION

Host immune protection against *N. meningitidis* invasive diseases depends almost entirely on a functional complement cascade culminating in bacteriolysis mediated by the membrane attack complex (6, 7), and the role of opsonophagocytosis against *N. meningitidis* is also appreciated to some extent (34, 35). Yet our study is the first in the context of *N. meningitidis* sepsis to demonstrate the devastating effect of the inflammatory branch of complement, which is mediated by complement split fragment C5a and its receptor C5aR1. We found that C5aR1 activation exaggerates the detrimental cytokine response to *N. meningitidis* sepsis. Most importantly, we could demonstrate that interference with complement-derived inflammation caused by pharmacological blockade of C5aR1 ameliorates inflammatory cytokine release in human whole-blood infection and leads to increased survival in experimental *N. meningitidis* sepsis. This opens up new therapeutic approaches to improve the outcome of patients with meningococcal sepsis, allowing the preservation of the bactericidal and opsonizing functions of complement vital for the host defense while specifically intercepting the detrimental actions of the C5a/C5aR1 axis.

Although generation of C5a was noted in rodent models of *N. meningitidis* (36, 37) and also in human meningitis patients (38), little attention has been paid to its functional contribution to IMD pathophysiology. Sprong et al. have demonstrated in human whole blood that blockade of C5a dampens granulocyte responses toward *N. meningitidis* (39), which is in good agreement with our results. Our data clearly demonstrate that *in vivo N. meningitidis* sepsis immunopathology is ameliorated when *C5ar1* is absent in mice (Fig. 3) or when C5aR1 is blocked (Fig. 7). With a decrease in susceptibility by an estimated factor of 5 to 10 (based on survival rates in *C5ar1*^−/−^ or C5aR1 antagonist-treated mice), the impact on IMD of C5a-derived inflammation has a smaller amplitude than but a direction clearly opposite from that of opsonization (*C3*^−/−^) and bacteriolysis (*Hc*°^/^°) in our *N. meningitidis* sepsis model.

One particularly striking finding is that *C5aR1*^−/−^ mice as well as WT mice treated with PMX205 displayed a significantly lower bacterial burden than the corresponding WT controls during the course of disease despite comparable levels of bacteremia at 3 h (Fig. 3C and 7B; see also Fig. S5B in the supplemental material). The fact that *C5aR1*^−/−^ mice mount a lower cytokine response to heat-inactivated *N. meningitidis* than WT mice as early as 3 h after injection suggests that the unbridled C5a/C5aR1 axis amplifies the initial inflammatory stimulus to the observed cytokine storm (Fig. 3D), which in turn dampens efficient pathogen clearance mechanisms of the host. This in turn may promote *N. meningitidis* replication in blood, leading to the observed higher bacterial burdens. The sepsis-induced “immune paralysis” reportedly affects primarily neutrophil functions such as chemotaxis and production of reactive oxygen species (40). Indeed, C5a appears to be centrally involved in the loss of neutrophil functions in the context of CLP sepsis models (17, 41).

Neutrophils are mobilized from bone marrow during *N. meningitidis* sepsis (42), and they are found in large numbers in cerebrospinal fluid of *N. meningitidis* meningitis patients. They can phagocytose *N. meningitidis* (34), but this mechanism is evidently not sufficient to control ongoing IMD. Furthermore, neutrophil activation causes collateral tissue damage, which may contribute significantly to IMD pathology. Thus, it is unclear whether the net effect of neutrophils on IMD pathology is beneficial or detrimental. We found that *C5ar1*^−/−^ neutrophils mount almost no oxidative burst in response to *N. meningitidis* (Fig. 4B) and a weaker degranulation response (Fig. 4C). Since *C5ar1*^−/−^ mice are less susceptible to *N. meningitidis* sepsis than WT mice (Fig. 3), we conclude that neutrophil oxidative burst and degranulation do not aid in *N. meningitidis* removal in this model. This notion is supported by studies demonstrating efficient mechanisms of *N. meningitidis* to avoid killing by reactive oxygen species *in vivo* (43, 44). Neutrophil adherence to lung endothelia can cause acute respiratory distress syndrome, a known complication during sepsis (45), and indeed, we observed high numbers of neutrophils in lungs of moribund WT mice (Fig. 4A), which suggests a partial involvement of neutrophils in the *N. meningitidis* sepsis pathophysiology. In contrast, neutrophils were important overall in the defense against *N. meningitidis*, as neutrophil-depleted mice succumbed to *N. meningitidis* sepsis at low inocula (Fig. 4F to I). Taking the data together, we conclude on the one hand that neutrophils are crucial against *N. meningitidis* sepsis and that this is independent of the oxidative burst response and degranulation and on the other hand that they contribute to disease pathophysiology.

One interesting side aspect of our study was the dissection of the relative contributions of the three major facets of complement activation—opsonization, inflammation, and lysis—to IMD. Our data suggest that, in experimental infections of mice, opsonization and bacteriolysis are both key to controlling the bacterial burden. Based on the survival rates reported in Fig. 1A, C5 deficiency increases susceptibility by ~100-fold and C3 deficiency increases it by at least 10-fold more, a difference that is presumably due to the added benefit of C3b-mediated opsonization of the meningococci. The importance of opsonophagocytosis was further corroborated by infections of mice depleted of their phagocyte populations, where monocyte/macrophage-depleted mice were much more susceptible to disease (Fig. 4F to I). However, it remains to be elucidated which monocyte/macrophage subpopulations are responsible for this dramatic effect. Neutrophils were an important determinant as well in this model but to a lesser extent than monocytes/macrophages. Given that *N. meningitidis* was not cleared *ex vivo* in mouse whole blood (Fig. 4E and 5C), we speculate that organ-resident phagocytes may be more effective in pathogen clearance than those circulating in blood, as recent findings suggest for *Streptococcus pneumoniae* (46).

Our finding that C5-deficient mice succumbed more easily to *N. meningitidis* sepsis in our model (Fig. 1A) is in apparent conflict with observations in humans with congenital defects in the terminal complement pathway (C6 to C9), who displayed an ameliorated course of disease resulting in a lower rate of mortality per episode of (often recurrent) IMD (7, 47). However, it is difficult to directly compare the course of disease in our animal model with that of human patients, since human patients receive immediate remedial attention and antibiotics, whereas the mice in our study did not. Since untreated human IMD is usually fatal (48, 49), we consider the mouse data to reflect the increased likelihood that a progressing invasive disease occurs after exposure to *N. meningitidis* rather than the course of disease itself.

Besides the capsule being its major virulence factor, *N. meningitidis* exploits various additional means to evade the complement system, such as recruitment of the complement regulators fH and C4BP, as well as secretion of the passenger domain of NalP which cleaves C3 (50, 51). These mechanisms aim at lowering the level of complement activation in response to *N. meningitidis* infection and might therefore also reduce detrimental effects of the C5a/C5aR1 axis. However, complement evasion allows *N. meningitidis* to establish a rapidly progressing infection, which in turn causes immunopathology, also involving the C5a/C5aR1 axis. Thus, the distinct effects of the complement evasion mechanisms of *N. meningitidis* on the role of C5a and C5aR1 during *N. meningitidis* sepsis merit further analysis.

Our data from *ex vivo* infections of human whole blood (Fig. 6) as well from *in vivo* mouse infections (Fig. 7) suggest the enticing possibility that C5aR1 might be a therapeutic target for adjunctive treatment of *N. meningitidis* sepsis. Several attempts to formulate adjunctive treatments aiming to reduce hyperinflammation have been made before, including administration of bactericidal/permeability-increasing protein (52) and anti-lipopolysaccharide (LPS) antibody treatment (53); however, these approaches failed to enhance *N. meningitidis* sepsis outcome in larger clinical trials (54, 55). Similarly, there seems to be no significant benefit of corticosteroid treatment of *N. meningitidis* meningitis (56) or sepsis (57). Thus, novel therapeutic approaches to specific interference with the uncontrolled inflammation during IMD are in dire need in order to enhance patient outcome. The fact that C5aR1 therapeutic inhibition performed with PMX205 treatment enhanced survival of the mice even when it was administered after infection (Fig. 7) makes C5aR1 an exciting candidate for adjuvant treatment. In fact, C5aR1 has been proposed as a therapeutic target to treat other inflammatory conditions, including inflammatory bowel disease (58), neurodegenerative diseases (59), ischemia-reperfusion injury (60), and general sepsis (61). Numerous studies showed a benefit of prophylactic blockade of C5a or C5aR1 prior to induction of an inflammatory insult in experimental inflammatory bowel disease (62), asthma (30, 63), or sepsis (64–67). In contrast, very few studies have demonstrated the utility of C5a/C5aR1 blockade in a therapeutic regimen. With respect to infectious diseases, therapeutic interference with the C5a/C5aR1 axis after induction of CLP sepsis was unsuccessful in two studies (66, 67) but demonstrated a benefit in another study (68); it seems likely that the complexity of microbial contaminations released into the bloodstream after intestinal perforation may impact the outcome of this approach. In contrast, the results of our study, focusing on sepsis caused by *N. meningitidis*, therefore represent an outstanding example of successful interference with the C5a/C5aR1 axis even after onset of the disease. Given that the human C5aR1-specific antagonist PMX53 has already undergone phase Ib/IIa clinical trials to treat psoriasis and rheumatoid arthritis and has passed safety and tolerability testing (69), this raises hope for a clear path for trials of PMX53, or other C5a-targeted therapeutics (70), to block the devastating outcome of invasive meningococcal disease.

## MATERIALS AND METHODS

### Bacteria.

*N. meningitidis* was grown on Columbia sheep agar plates (BioMérieux) at 37°C and 5% CO_2_ in a water-saturated atmosphere. Strain MC58 was used for most experiments and belongs to serogroup B (type B:15:P1.7,16-2:F1-5; ST-74, ST-32 complex) (71). Its coisogenic mutants, MC58Δ*csb* (lacking capsule) and MC58Δ*csb*-GFP (expressing green fluorescent protein), and their respective plasmid constructs were described elsewhere (72, 73). Further information on the *N. meningitidis* strains used in this study is available in Table S1 in the supplemental material.

10.1128/mBio.01755-17.9 TABLE S1  *N. meningitidis* strains used in this study. Download TABLE S1, PDF file, 0.1 MB. Copyright © 2018 Herrmann et al. 2018 Herrmann et al. This content is distributed under the terms of the Creative Commons Attribution 4.0 International license.

### Animals.

Animals were housed with a 12-h-bright/12-h-dark cycle at 22 to 24°C and 55% ± 10% relative humidity and hygiene standards in agreement with specified pathogen-free conditions as tested quarterly following recommendations of the Federation for European Laboratory Animal Science Associations (FELASA). Mice were always negative for all relevant viruses, parasites, and bacteria (including *Helicobacter* spp.). Mice had *ad libitum* access to standard diet and water, and cages were equipped with enrichment. All mouse strains used were congenic, with C57BL/6J as the genetic background. Wild-type (WT) mice were obtained from Charles River or Envigo (formerly Harlan). B6.129S4-*C5ar1*^tm1Cge^/J (C5aR1^−/−^) mice and B6.129S4-C3^tm1Crr^/J (*C3*^−/−^) mice were provided by A. Klos, Hannover Medical School. B6.FVB-Hc° (*Hc*°^/^°) mice lacking complement component C5 were generated in this work. Mouse strains were held within the same rooms, and litter exchanges were performed 2 weeks before experiments in order to equalize their microflora.

### Mouse sepsis model.

For sepsis induction, 6-to-8-week-old male mice were restricted in a supine position and 200 µl of bacterial inocula prepared as described below was intraperitoneally injected along with 200 µl of iron dextran (Sigma) diluted in sterile isotonic saline solution corresponding to 2 mg free iron. Mice were scored every 6 h by applying the following scheme: >3% to 5% loss of body weight, score of 1; ≥5% to 10% loss of body weight, score of 5; ≥10% to 20% loss of body weight, score of 10; ≥20% loss of body weight, score of 20; mildly aberrant general condition, score of 1; dull coat, mild lack of grooming, mildly hunched posture, score of 5; dirty coat, smudgy eyes, partially closed eyes, hunched posture, mild dehydration, dirty anus, diarrhea, score of 10; severely hunched posture, severe dehydration, tremors, spasms, inability to rise from lateral position, ataxia, score of 20; mildly aberrant behavior, score of 1; reduced movements, score of 5; isolation from group, lethargy, score of 10; unconscious, no defensive behavior upon picking up, score of 20; mild clinical symptoms, score of 1; breathing frequency increased or decreased 30%, score of 10; breathing frequency increased or decreased 50%, circulatory disturbance, score of 20. Following regulations of the German and the Canadian Animal Welfare Laws, animals were not let to die on their own but were sacrificed upon reaching the humane end point criteria (ataxia, tremors, and inability to rise from lateral position).

To prepare *N. meningitidis* inocula, overnight growth of *N. meningitidis* was transferred onto blood agar plates and incubated for 4 h as described above. Growth was resuspended in brain heart infusion broth (BHI) and adjusted to the appropriate density, with an optical density at 600 nm (OD_600_) of 1.0 yielding 1.5 × 10^9^ CFU/ml. Inoculum accuracy was confirmed by dilution plating. For heat-inactivated inocula, CFU count testing was done before heating the inoculum to 65°C for 30 min.

### C5aR1 antagonists.

For *in vivo* blockade of mouse C5aR1, cyclic peptide PMX205 was used as outlined above. Synthesis of PMX205 (cyclic hexapeptide hydro-cinnamate-[l-ornithine-proline-d-cyclohexylalanine-tryptophan-arginine]) is described elsewhere (62). For *ex vivo* blockade of human C5aR1, cyclic peptide PMX53 (Tocris Bioscience) and nonpeptide compound W-54011 (Merck Millipore) were used at the indicated concentrations.

### *In vivo* depletion and C5aR1 antagonist treatment.

Where indicated, mice were treated as follows. To deplete monocytes and macrophages, mice were subjected to i.p. injection with 200 µl clodronate liposomes (Clodronate Liposomes) at day −3 and again at 12 h prior to infection. To deplete neutrophil granulocytes, mice were subjected to i.p. injection with 250 µg of clone RB6-8C5 rat anti-neutrophil antibody at −24 h, 0 h, 24 h, and 48 h. Control animals received 200-µl PBS injections at the same time points.

To block C5aR1, mice were subjected to i.p. injection with the C5aR1 antagonist PMX205 at 3 mg/kg of body weight in 100 µl of 5% glucose solution either at −12 h, −6 h, 0 h, 12 h, and 24 h (“pretreatment”) or at 4 h, 9 h, 15 h, and 24 h (“posttreatment”), whereas control animals received 5% glucose solution at the same time points.

### Assessment of C3b deposition on *N. meningitidis*.

In 96-well microtiter plates, 50-µl volumes of a heat-inactivated suspension of *N. meningitidis* (strain MC58) at an OD_600_ of 0.1 were allowed to dry. Plates were washed thrice with PBS–0.05% Tween 20 and blocked with PBS–5% bovine serum albumin (BSA). Where indicated, protein G-purified rabbit anti-*N. meningitidis* antibody was added at 45 µg/ml to PBS–1% BSA and the reaction mixture was incubated for 30 min. After washing, freshly obtained lepirudinized mouse plasma (see below) was added at 50 µl per well at a 1:10 dilution to HEPES-based KBR buffer (Virion-Serion) and incubated at 37°C for 30 min. In negative-control wells, 5 mM EDTA was added. After washing was performed, wells were fixed with 4% paraformaldehyde (PFA), washed again, and incubated using goat anti-C3 antibody (Complement Technologies) and donkey anti-goat horseradish peroxidase (HRP) conjugate (Jackson ImmunoResearch), followed by addition of TMB (3,3′,5,5′-tetramethylbenzidine) substrate (Pierce) for colorimetry.

### *N. meningitidis* viability assay.

To test bactericidal activity of PMX205, *N. meningitidis* strain MC58 was prepared as outlined above and incubated at 10^4^ CFU per ml in a total volume of 50 µl pooled mouse serum supplemented with the indicated concentrations of PMX205 for 1 h at 37°C in triplicate. After dilution plating and overnight incubation performed as outlined above, colonies were enumerated.

### Immunofluorescence microscopy.

Paraffin sections (5 μm) of organs from infected or control animals were deparaffinized and subjected to antigen retrieval by boiling in 10 mM citrate buffer at pH 6.0 before equilibration in PBS and blocking in PBS with 5% BSA (AppliChem), 5% goat serum (Sigma-Aldrich), and 0.5% Fc-block (Becton, Dickinson) were performed. Neutrophils were stained with polyclonal rabbit anti-neutrophil elastase antibody (Abcam, Inc.) followed by goat anti-rabbit Cy3 (Jackson ImmunoResearch). *N. meningitidis* was then stained using fluorescein isothiocyanate (FITC)-labeled anti-*N. meningitidis* polyclonal rabbit antibody. The rabbit anti-*N. meningitidis* antibody raised against strain MC58 was described elsewhere (74). Sections were counterstained with DAPI (4′,6-diamidino-2-phenylindole) and mounted with Fluoroshield (Sigma-Aldrich). Immunofluorescence microscopy was carried out on a BZ-9000 Biorevo digital microscope (Keyence) at ×60 and ×100 magnification using the full-focus function to collapse z-stacks in BZ II Viewer software for image capture and the black balance function and channel merge function in BZ II Analyzer software.

### ELISAs.

Mouse C3a enzyme-linked immunosorbent assays (ELISAs) were carried out using rat anti-mouse C3a clone I87-1162 antibody (Becton, Dickinson) as the capture antibody and biotin-rat anti-mouse C3a clone I87-419 antibody (Becton, Dickinson) as the detection antibody and avidin-peroxidase (Jackson ImmunoResearch) and TMB substrate (Pierce) for colorimetry. Dilutions of zymosan-activated mouse serum were used as the standard, with 1 ml containing 1,000 au of mouse C3a (mC3a). Mouse C5a ELISA was carried out using rat anti-mouse C5a clone I52-1486 antibody (Becton, Dickinson) as the capture antibody and biotin-rat anti-mouse C5a clone I52-278 antibody (Becton, Dickinson) as the detection antibody. As standards, dilutions of purified recombinant mouse C5a (R&D Systems) were used. Mouse CXCL-1, IL-6, and TNF-α and human IL-8 levels were measured using DuoSet ELISA kits (R&D Systems). Human IL-1β, TNF-α, and IL-6 levels were measured using LegendPlex immunoassay (BioLegend).

### Whole-blood models.

For mouse whole-blood models, animals were euthanized by CO_2_ asphyxiation and cardiac puncture was performed with 20 µl per ml of blood of lepirudin (Refludan, Bayer), which does not interfere with complement (24), as an anticoagulant. Human whole blood was obtained from healthy adult volunteers without signs of chronic or acute disease or inflammatory conditions by venipuncture using hirudin Monovettes (Sarstedt) to avoid clotting without impairing complement integrity. Blood samples were used immediately for experiments. Where indicated, C5aR1 antagonists were added immediately to the blood followed by incubation for 10 min prior to infection. For infections, bacterial inocula were adjusted in PBS, with 1 part added to 10 parts of blood. Samples were incubated rotating over top at 37°C.

### Measurement of *N. meningitidis* phagocytosis.

Mouse whole blood was inoculated with 10^7^ CFU/ml of *N. meningitidis* MC58Δ*csb*-GFP for 1 h with rotation at 37°C. A noncapsulated strain was chosen in order to obtain a clear signal in this assay, since capsule expression lowers C3b deposition and phagocytosis. Samples were then transferred on ice for 1 h with allophycocyanin (APC) anti-Ly6G clone 1A8 (BioLegend) to stain neutrophils. After hypotonic lysis of erythrocytes with 168 mM NH_4_Cl–10 mM KHCO_3_–0.125 mM EDTA for 5 min, cell pellets were washed in PBS, fixed in 4% PFA, and subjected to flow cytometry on a FACSCalibur instrument (Becton, Dickinson). Phagocytosis was assessed as the increase in GFP fluorescence (channel FL1-H) of the neutrophils gated as Ly6G^hi^. For assays performed with human blood, MC58-GFP (encapsulated) was used and the PMN population identified by forward scatter/side scatter (FSC/SSC) only. Data were analyzed and graphed using FlowJo v10.

### Measurement of oxidative burst.

Oxidative burst was measured in whole blood using DHR123 (Sigma-Aldrich) at a final concentration of 20 µg/ml. Blood was incubated with DHR123 and bacteria or PMA as a positive control for 1 h at 37°C. Samples were then incubated on ice for 1 h with APC anti-Ly6G clone 1A8 (BioLegend) to stain mouse neutrophils. After erythrocyte lysis and fixation performed as described above, cells were analyzed by flow cytometry on a FACSCalibur instrument (Becton, Dickinson). Mouse neutrophils were gated as Ly6G^hi^ cells, and human cells were gated by FSC/SSC. The oxidative burst was assessed as fluorescence of DHR123 in channel FL1-H. Data were analyzed and graphed using FlowJo v10.

### Flow cytometry for surface markers.

To assess surface localization of C5aR1, samples of mouse whole-blood samples infected with 10^7^ CFU of *N. meningitidis* or controls treated with PBS alone were incubated at 37°C for 1 h and then transferred to ice. Samples were incubated either with FITC-rat anti-mouse Ly6G clone 1A8 (BioLegend) to stain neutrophils along with APC-rat anti-mouse C5aR clone 20/70 (BioLegend) or with APC-rat anti-mouse Ly6G clone 1A8 (BioLegend) along with FITC-rat anti-CD11b clone M1/70 (BioLegend) for 1 h on ice. Then, erythrocytes were lysed as outlined above, fixed with 4% PFA, and analyzed on a FACSCalibur instrument (Becton, Dickinson). For measurement of levels of surface CD11b with human blood, the same antibody (clone M1/70) as that used for mice was used and the PMN population was identified by FSC/SSC only. Flow cytometry data were analyzed and graphed using FlowJo v10 (FlowJo, LLC).

### Ethics statement.

Animal experiments were approved either by the Animal Ethics Review Committee of the University of Toronto (permits 20008007 and 20008657), which is subject to ethical and legal requirements under the province of Ontario’s Animals for Research Act and the federal Council on Animal Care (CCAC), or by the Government of Lower Franconia, adhering to the German Animal Welfare Law (permit 55.2-2531.01-14/14). All efforts were made to minimize suffering.

Human blood samples were obtained after written informed consent was obtained prior to blood collection. The use of human material and the study design were approved by the Ethical Review Committee of the University Clinic of Würzburg (permit 181/16-ge), adhering to the Helsinki Declaration.
