# Supplementary figures and images for: Complement C5a Receptor 1 Exacerbates the Pathophysiology of N. meningitidis Sepsis and Is a Potential Target for Disease Treatment
Source: mBio. 2018 Jan 23;9(1):e01755-17. doi: 10.1128/mBio.01755-17 (PMC5784250; doi:10.1128/mBio.01755-17)

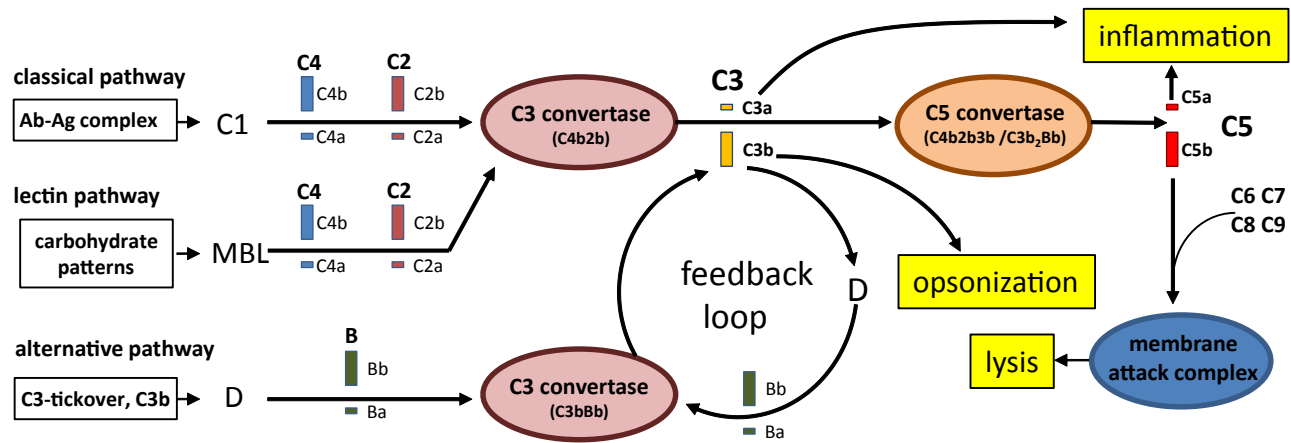

Figure S1: Schematic of the complement cascade

Supplement: FIG S1 [file mbo001183685sf1.pdf]
