# Supplementary material for: Complement C5a Receptor 1 Exacerbates the Pathophysiology of N. meningitidis Sepsis and Is a Potential Target for Disease Treatment
Source: mBio. 2018 Jan 23;9(1):e01755-17. doi: 10.1128/mBio.01755-17 (PMC5784250; doi:10.1128/mBio.01755-17)
Supplement: FIG S2 [file mbo001183685sf2.pdf]

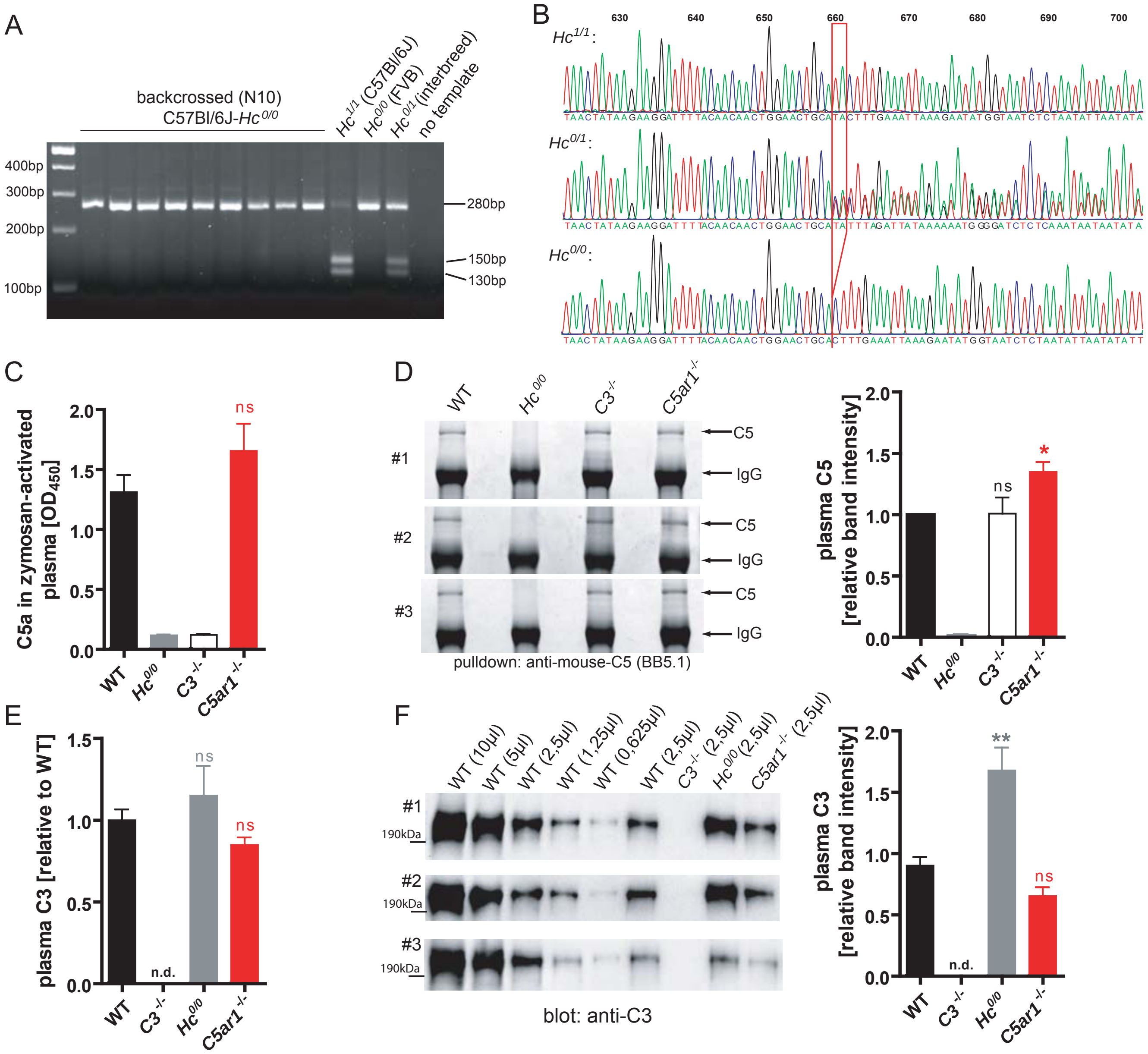

Figure S2: Characterization of $Hc^{0/0}$ mice. A: Genotyping of litters of founders of the B6.FVB-Hc^0 line ($Hc^{0/0}$) after ten generations of backcrossing the Hc^0 allele onto C57Bl/6J background. The 280 bp amplicon of the marker associated with the wild type *Hc* gene, which encodes mouse C5, is cleaved by HindIII, whereas that associated with Hc^0 is not. **B:** Sequencing of the *Hc* gene portion containing the two-basepair gap (TA at position 660) responsible for premature termination of transcription and loss of C5 in the Hc^0 allele. Note the sequence disalignment starting at base 660 in heterozygous ($Hc^{0/1}$) sample. **C:** Measurement of C5a in lepirudinized mouse plasma after activation with 20 mg/ml zymosan. No C5a generation is observed in either C3 or C5 deficient plasma samples whereas a similar signal is observed in WT and $C5ar1^{-/-}$ plasma samples. Data plotted are means ± SEM of samples obtained from three animals per genotype. **D:** Coomassie-stained gels after non-reducing SDS-PAGE of C5 pulldown from mouse EDTA-plasma samples using anti-mouse-C5 antibody (clone BB5.1) coupled to protein G coated magnetic beads. Three independent experiments were conducted and relative band intensities analyzed. Plotted are means ± SEM. **E:** Plasma levels of C3 in mouse strains by semi-quantitative ELISA. Plotted are means ± SEM of samples from three animals per genotype. **F:** Western blotting of EDTA-plasma samples in mouse strains and quantification by band intensities. Data are means ± SEM of three independent runs. All panels: ns, not significant; *, $P < 0.05$ in one-way ANOVA applying Dunnett's *post hoc* test with WT as control group.
