# Supplementary material for: Complement C5a Receptor 1 Exacerbates the Pathophysiology of N. meningitidis Sepsis and Is a Potential Target for Disease Treatment
Source: mBio. 2018 Jan 23;9(1):e01755-17. doi: 10.1128/mBio.01755-17 (PMC5784250; doi:10.1128/mBio.01755-17)
Supplement: FIG S3 [file mbo001183685sf3.pdf]

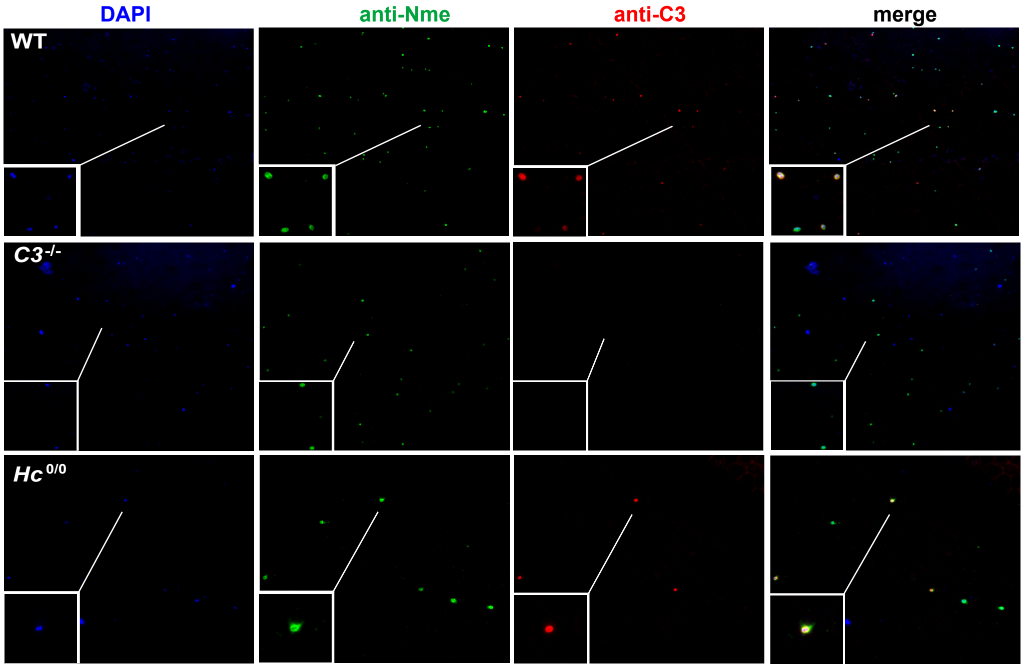

Figure S3: Representative immunofluorescence images for graph in Fig.1B. Bloodsmears of mice (indicated genotypes) 12 h after infection were analyzed by immunofluorescence microscopy after staining with DAPI, rabbit-anti-*Nme* (Alexa488 channel) and anti-C3 (Cy3 channel) at 60x magnification.
