# Supplementary material for: Complement C5a Receptor 1 Exacerbates the Pathophysiology of N. meningitidis Sepsis and Is a Potential Target for Disease Treatment
Source: mBio. 2018 Jan 23;9(1):e01755-17. doi: 10.1128/mBio.01755-17 (PMC5784250; doi:10.1128/mBio.01755-17)
Supplement: FIG S4 [file mbo001183685sf4.pdf]

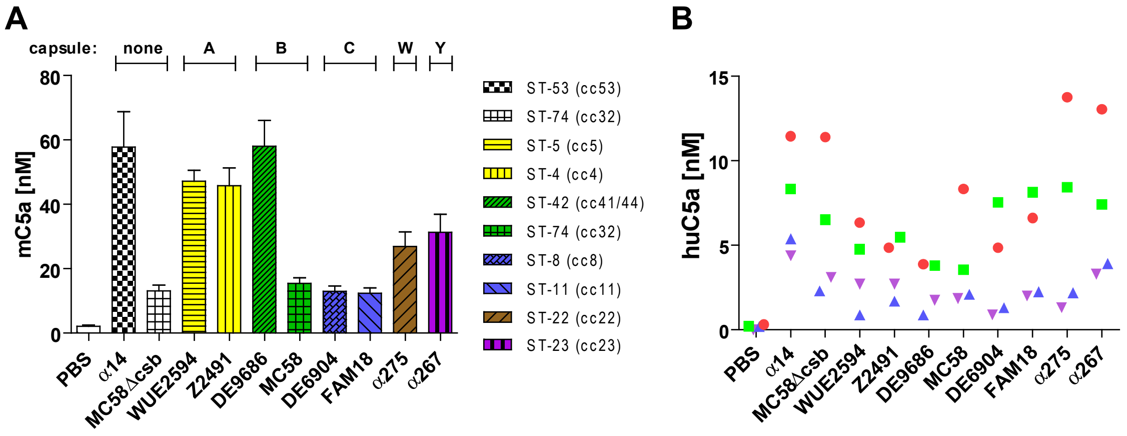

Figure S4: C5a-liberation in whole blood models of mouse and human by different *Nme* strains. A: C5a release in in *ex vivo* infection of lepirudin whole mouse blood with 10^{8}CFU/ml of various *Nme* strains covering most relevant serogroups and sequence types as indicated in legend (means + SEM; n = 3). **B:** Same as for panel A, but with human whole blood (n = 4 individual donors, indicated by individual symbols).
