# Supplementary material for: Complement C5a Receptor 1 Exacerbates the Pathophysiology of N. meningitidis Sepsis and Is a Potential Target for Disease Treatment
Source: mBio. 2018 Jan 23;9(1):e01755-17. doi: 10.1128/mBio.01755-17 (PMC5784250; doi:10.1128/mBio.01755-17)
Supplement: FIG S5 [file mbo001183685sf5.pdf]

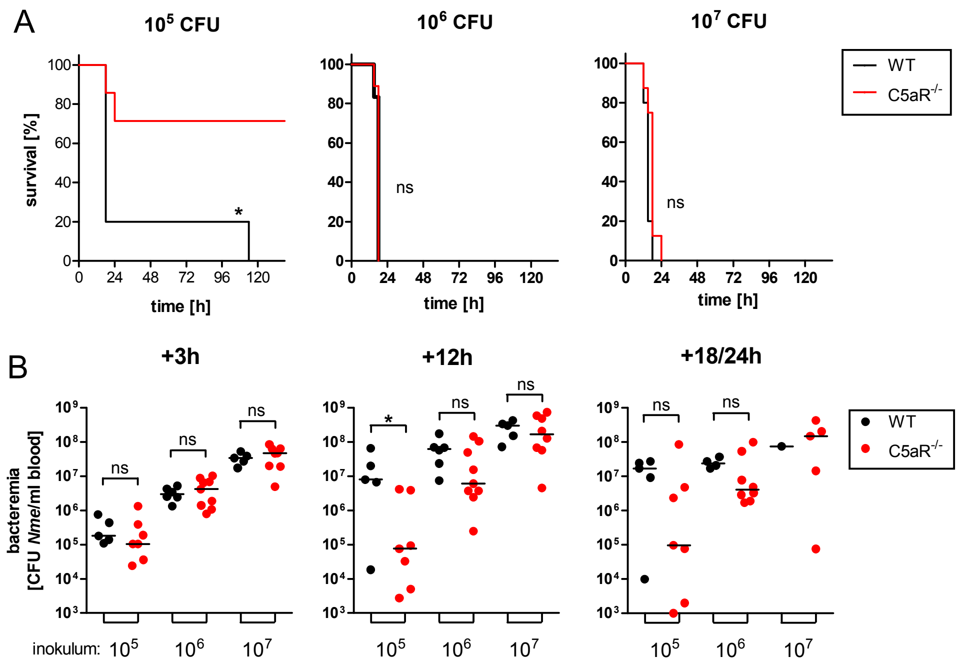


**Figure S5: Survival and bacteremia in WT versus *C5ar1*$^{-/-}$ mice after infection with different inocula of *Nme*. A:** Survival curves of WT and *C5aR1*$^{-/-}$ mice after intraperitoneal infection with $10^5$, $10^6$ or $10^7$ CFU of *Nme* strain MC58 as indicated above graphs. ns, not significant; *, $P < 0.05$ in Mantel-Cox analysis. **B:** Bacteremia in blood of the same mice as in above graph assessed at 3 h, 12 h and 18/24 h after infection. 18 h values were included in the 18/24 h data set from those mice which did not survive until 24 h. Plotted are values of individual mice, lines indicate median. ns, not significant; *, $P < 0.05$ in unpaired, two-tailed Mann-Whitney test comparing WT vs. *C5aR*$^{-/-}$ separately for each inokulum and time point. Note that mice in the cohorts infected with $10^5$ CFU are identical to a subset of mice in Fig. 3 A,B and are plotted here as comparison from within the same experiment.
