# Supplementary material for: Complement C5a Receptor 1 Exacerbates the Pathophysiology of N. meningitidis Sepsis and Is a Potential Target for Disease Treatment
Source: mBio. 2018 Jan 23;9(1):e01755-17. doi: 10.1128/mBio.01755-17 (PMC5784250; doi:10.1128/mBio.01755-17)
Supplement: FIG S6 [file mbo001183685sf6.pdf]

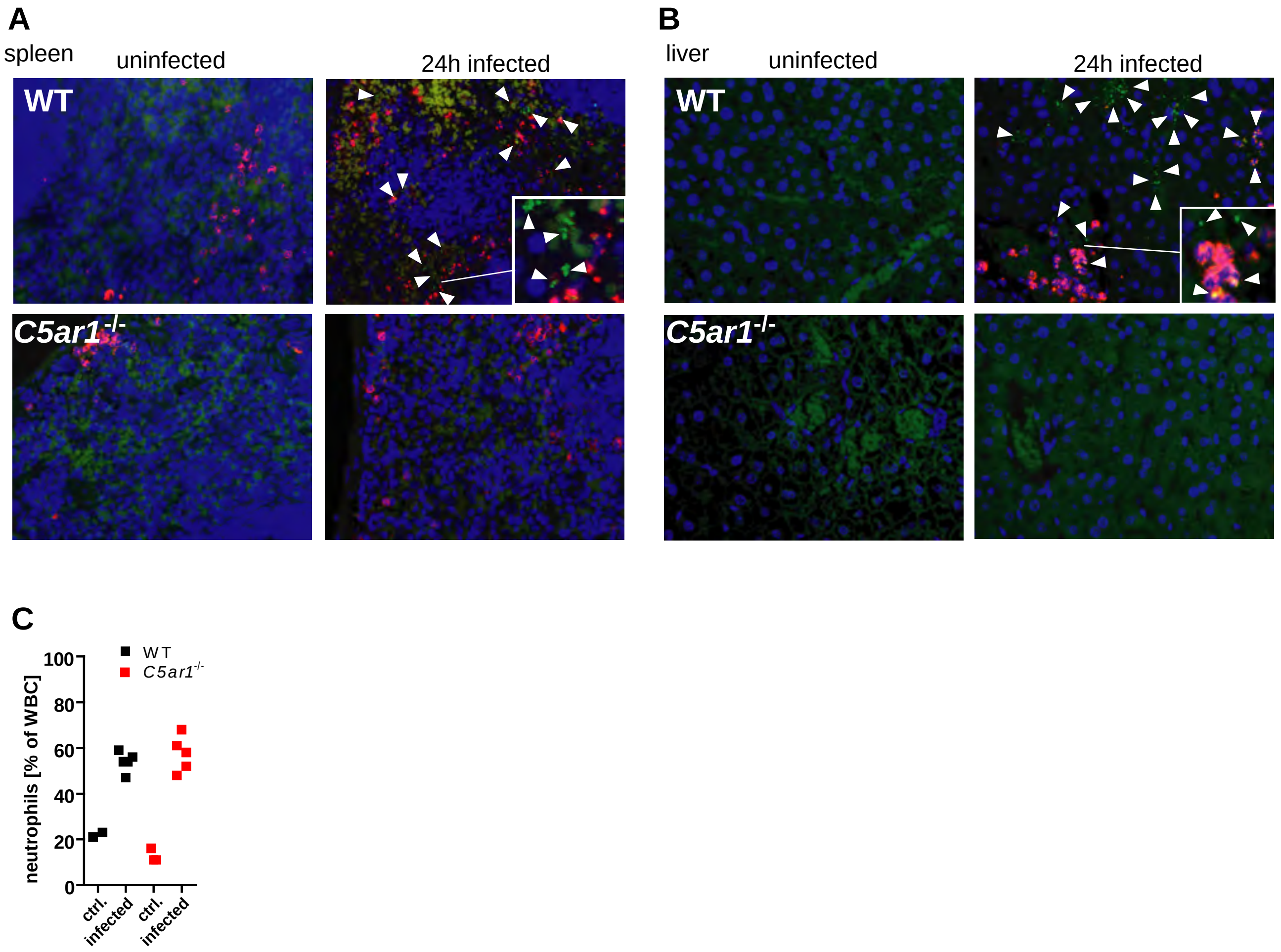


**Figure S6: Neutrophil mobilization during *Nme* sepsis. A, B:** Immunofluorescence microscopy images of spleen (A) and liver (B) of infected WT or *C5ar1*$^{-/-}$ mice. Staining was done as described in Figure 4A. **C:** Neutrophil numbers in blood (expressed as percentage of white blood cells) as assessed by differential counting of bloodsmears from infected and non-infected WT versus *C5ar1* $^{-/-}$mice.
