# Supplementary material for: Complement C5a Receptor 1 Exacerbates the Pathophysiology of N. meningitidis Sepsis and Is a Potential Target for Disease Treatment
Source: mBio. 2018 Jan 23;9(1):e01755-17. doi: 10.1128/mBio.01755-17 (PMC5784250; doi:10.1128/mBio.01755-17)
Supplement: FIG S7 [file mbo001183685sf7.pdf]

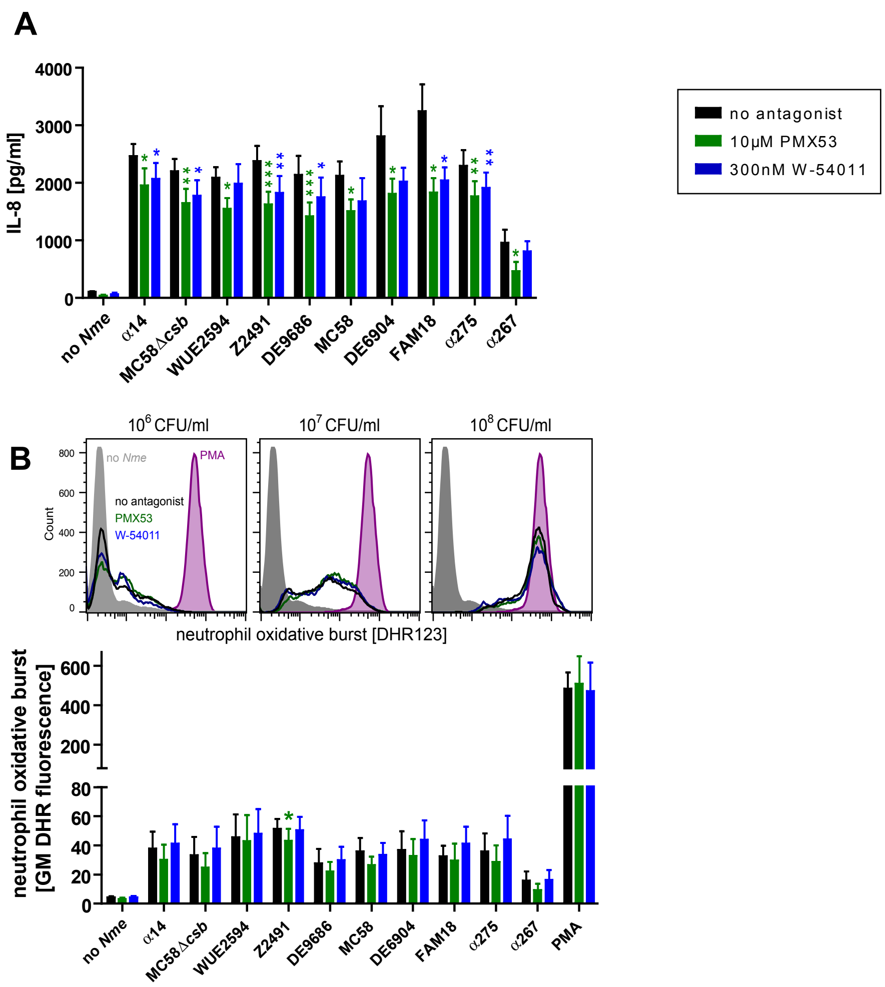


**Figure S7: IL-8 secretion and oxidative burst in human whole blood infected with various *Nme* strains. A:** IL-8 (mean ± SEM) in human whole blood infection (n = 4 donors) with $10^6$ CFU/ml of *Nme* strains (indicated below x-axis) in presence of PMX53 (10 µM) or W-54011 (300 nM) at 90 min. **B:** Neutrophil oxidative burst after 1h infection of whole human blood. Representative histograms show results for infection with $10^6$, $10^7$ or $10^8$ CFU/ml of *Nme* MC58 in presence or absence of C5aR1 antagonists. Bar graph below shows results from infections with $10^7$ CFU/ml of *Nme* strains (indicated below x-axis) as mean fluorescence intensity (MFI) ± SEM (n = 4). *,**, ***: P < 0,05, 0.01 or 0.005, respectively, in repeated measures ANOVA (matched observations per individual donor), applying Dunnett's *post hoc* test. If no indication is given, differences were not significant.
