# Supplementary material for: Complement C5a Receptor 1 Exacerbates the Pathophysiology of N. meningitidis Sepsis and Is a Potential Target for Disease Treatment
Source: mBio. 2018 Jan 23;9(1):e01755-17. doi: 10.1128/mBio.01755-17 (PMC5784250; doi:10.1128/mBio.01755-17)
Supplement: FIG S8 [file mbo001183685sf8.pdf]

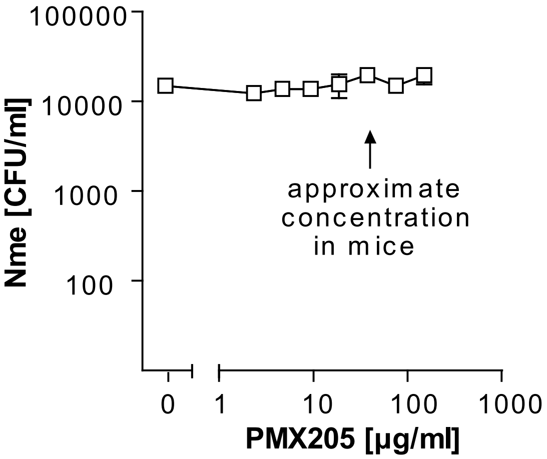

Fig. S8: No bactericidal activity of PMX205 towards *Nme*. *Nme* viability was assayed by incubation of *Nme* MC58 at 37°C for 1 h in mouse serum with indicated concentrations of PMX205 (mean ± SEM; n = 3) before dilution plating.
