# Supplementary material for: Complement C5a Receptor 1 Exacerbates the Pathophysiology of N. meningitidis Sepsis and Is a Potential Target for Disease Treatment
Source: mBio. 2018 Jan 23;9(1):e01755-17. doi: 10.1128/mBio.01755-17 (PMC5784250; doi:10.1128/mBio.01755-17)
Supplement: TABLE S1 [file mbo001183685st1.pdf]

Table S1: *Nme* strains

strain	sero-group	sequence type	clonal complex	source	country	year	reference
**MC58**	B	ST-74	ST-32	disease	UK	1985	(McGuinness et al., 1991)
**MC58Δ*csb***	-	ST-74	ST-32	-	-	-	(Lappann et al., 2006)
**MC58Δ*csb*-GFP**	-	ST-74	ST-32	-	-	-	(Lappann et al., 2006)
**α14**	*cnl*	ST-53	ST-53	carriage	Germany	1999	(Claus et al., 2005)
**WUE2495**	A	ST-5	ST-5	disease	Germany	1991	(Lewis et al., 2010)
**Z2491**	A	ST-4	ST-4	disease	Gambia	1983	(Crowe et al., 1989)
**DE9686**	B	ST-42	ST-41/44	disease	Germany	2004	(Elias et al., 2010)
**DE6904**	C	ST-8	ST-8	disease	Germany	2002	(Brehony et al., 2007)
**FAM18**	C	ST-11	ST-11	disease	USA	1980	(Bentley et al., 2007)
**α275**	W	ST-22	ST-22	carriage	Germany	2000	(Claus et al., 2005)
**α269**	Y	ST-23	ST-23	carriage	Germany	2000	(Claus et al., 2005)

cnl: capsule null locus

References:

Bentley, S.D., G.S. Vernikos, L.A. Snyder, C. Churcher, C. Arrowsmith, T. Chillingworth, A. Cronin, P.H. Davis, N.E. Holroyd, K. Jagels, M. Maddison, S. Moule, E. Rabbinowitsch, S. Sharp, L. Unwin, S. Whitehead, M.A. Quail, M. Achtman, B. Barrell, N.J. Saunders, and J. Parkhill. 2007. Meningococcal genetic variation mechanisms viewed through comparative analysis of serogroup C strain FAM18. *PLoS genetics* 3:e23.

Brehony, C., K.A. Jolley, and M.C. Maiden. 2007. Multilocus sequence typing for global surveillance of meningococcal disease. *FEMS microbiology reviews* 31:15-26.

Claus, H., M.C. Maiden, D.J. Wilson, N.D. McCarthy, K.A. Jolley, R. Urwin, F. Hessler, M. Frosch, and U. Vogel. 2005. Genetic analysis of meningococci carried by children and young adults. *The Journal of infectious diseases* 191:1263-1271.

Crowe, B.A., R.A. Wall, B. Kusecek, B. Neumann, T. Olyhoek, H. Abdillahi, M. Hassan-King, B.M. Greenwood, J.T. Poolman, and M. Achtman. 1989. Clonal and variable properties of *Neisseria meningitidis* isolated from cases and carriers during and after an epidemic in The Gambia, West Africa. *The Journal of infectious diseases* 159:686-700.

Elias, J., L.M. Schouls, I. van de Pol, W.C. Keijzers, D.R. Martin, A. Glennie, P. Oster, M. Frosch, U. Vogel, and A. van der Ende. 2010. Vaccine preventability of meningococcal clone, Greater Aachen Region, Germany. *Emerging infectious diseases* 16:465-472.

Lappann, M., J.A. Haagensen, H. Claus, U. Vogel, and S. Molin. 2006. Meningococcal biofilm formation: structure, development and phenotypes in a standardized continuous flow system. *Molecular microbiology* 62:1292-1309.

Lewis, L.A., J. Ngampasutadol, R. Wallace, J.E. Reid, U. Vogel, and S. Ram. 2010. The meningococcal vaccine candidate neisserial surface protein A (NspA) binds to factor H and enhances meningococcal resistance to complement. *PLoS pathogens* 6:e1001027.

McGuinness, B.T., I.N. Clarke, P.R. Lambden, A.K. Barlow, J.T. Poolman, D.M. Jones, and J.E. Heckels. 1991. Point mutation in meningococcal por A gene associated with increased endemic disease. *Lancet* 337:514-517.
